# Comparison and transfer testing of multiplex ligation detection methods for GM plants

**DOI:** 10.1186/1472-6750-12-4

**Published:** 2012-01-19

**Authors:** Gabriella Ujhelyi, Jeroen P van Dijk, Theo W Prins, Marleen M Voorhuijzen, AM Angeline Van Hoef, Henriek G Beenen, Dany Morisset, Kristina Gruden, Esther J Kok

**Affiliations:** 1 CFRI - Central Food Research Institute, Herman Ottó út 15. H-1022, Budapest, Hungary; 2 RIKILT - Institute of Food Safety (WUR), Akkermaalsbos 2, 6708 WB, Wageningen, the Netherlands; 3 Laboratory of Phytopathology (WUR), Droevendaalsesteeg 1, 6708 PB, Wageningen, the Netherlands; 4 NIB - Department of Biotechnology and Systems Biology, National Institute of Biology, Večna pot 111, 1000 Ljubljana, Slovenia

## Abstract

**Background:**

With the increasing number of GMOs on the global market the maintenance of European GMO regulations is becoming more complex. For the analysis of a single food or feed sample it is necessary to assess the sample for the presence of many GMO-targets simultaneously at a sensitive level. Several methods have been published regarding DNA-based multidetection. Multiplex ligation detection methods have been described that use the same basic approach: i) hybridisation and ligation of specific probes, ii) amplification of the ligated probes and iii) detection and identification of the amplified products. Despite they all have this same basis, the published ligation methods differ radically. The present study investigated with real-time PCR whether these different ligation methods have any influence on the performance of the probes. Sensitivity and the specificity of the padlock probes (PLPs) with the ligation protocol with the best performance were also tested and the selected method was initially validated in a laboratory exchange study.

**Results:**

Of the ligation protocols tested in this study, the best results were obtained with the PPLMD I and PPLMD II protocols and no consistent differences between these two protocols were observed. Both protocols are based on padlock probe ligation combined with microarray detection. Twenty PLPs were tested for specificity and the best probes were subjected to further evaluation. Up to 13 targets were detected specifically and simultaneously. During the interlaboratory exchange study similar results were achieved by the two participating institutes (NIB, Slovenia, and RIKILT, the Netherlands).

**Conclusions:**

From the comparison of ligation protocols it can be concluded that two protocols perform equally well on the basis of the selected set of PLPs. Using the most ideal parameters the multiplicity of one of the methods was tested and 13 targets were successfully and specifically detected. In the interlaboratory exchange study it was shown that the selected method meets the 0.1% sensitivity criterion. The present study thus shows that specific and sensitive multidetection of GMO targets is now feasible.

## Background

The adoption of crops that are genetically modified organisms (GMOs) has continuously increased over the last decade with 148 million hectares grown in 2010 worldwide [1]. Because of the increasing number of GM crops, the analysis of an individual food or feed sample for the potential presence of GMOs becomes more complex, time-consuming and expensive. To overcome these problems it is necessary to develop a method which can identify many GMO-derived DNA targets in a single experiment, at a sensitive level, reducing both cost and analysis time. The potential presence of unauthorized GM crops makes the situation even more complicated [2,3].

Currently, the most common method to detect and identify GMOs in food and feed products is real-time polymerase chain reaction (PCR). For most targets this method has a limit of detection (LOD) of 0.1% or less. In the scientific literature, different multiplex GMO detection methods have been described but various problems with detection level and specificity have been reported. Ligation-based systems seem very promising approaches to detect GMOs in a multiplex setting in a sensitive and specific way.

**Figure 1:**
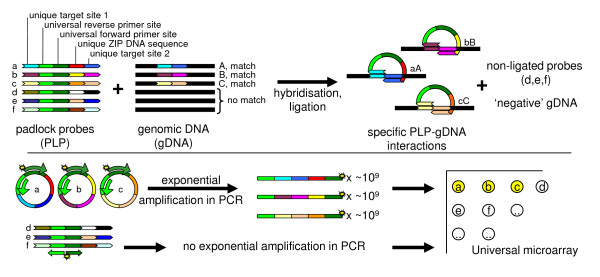
**Scheme of the padlock ligation detection procedure**. A mix of linear padlock probes can hybridize to their genomic counterparts, after which the juxtaposed ends are ligated to form a circular molecule. Only ligated, circular molecules are amplified by subsequent PCR with a universal forward and Cy3-labelled reverse primer. Non-ligated probes will not be amplified as the primer sites point away from each other. Each probe contains a unique DNA sequence (cZIP-code). After PCR the products are visualized by hybridization of the Cy3-labelled molecule on a microarray via a homologous ZIP sequence.

Ligation was one of the first tools in the hands of molecular biologists for cloning and DNA manipulation and has played a major role in explanation of gene functions. It was also found that ligation can be used for detection of specific DNA sequences [4]. During the 1990s several ideas and theories were examined for making ligation detection more sensitive and applicable for multiplex detection. One of the resulting strategies used so-called padlock probes (PLPs). PLPs were designed to be linear with the ligation sites at the extremities. The PLP was shown to be circularized after ligation [5] and with this method up to 10,000 DNA targets were detected simultaneously in a human setting [6]. In the area of single-nucleotide polymorphism (SNP) detection of up to thousands of targets has been reached [7]. A PLP usually contains universal primer sites for PCR amplification and a universal microarray can be used for detection and identification (Figure 1). Such a padlock system was adapted to detect and identify (GMO) crops [8,9].

In a tenplex PLP experiment different genomic targets in GTS 40-3-2 soy, MON1445 cotton and Bt176 maize were detected down to at least 1% [8]. The PLP system can be used not just for GMO detection but also for other nucleic acid experiments. It was for instance used for SNP-based genotyping in allohexaploid wheat [10].

Other ligation based techniques have been developed to detect GMOs as well. One of these uses two separate " bipartite ligation" probes for each target. After the amplification of the targets the detection can be performed either by capillary electrophoresis or by microarray hybridization. This kind of ligation-dependent probe amplification (LPA) system was used by Moreano et al. [11] to detect several targets. In their study two endogenous targets and two event specific junction regions were detected simultaneously. GMO maize DNA (0.1%) was detected in the presence of 5% GM soy DNA and vice versa. This group improved the above-mentioned LPA technique for more targets [12]. This LPA technique was also used for simultaneous detection of 10 allergens [13]. Holck et al. [14] developed a nineplex ligation-dependent probe amplification method for detection of seven GM maize events, one GM maize construct and one endogenous maize reference gene.

A so-called SNPlex method, which used also two separate probes, has also been tested for GM detection by Chaouachi et al. [15]. Probes in this paper contained universal primer sites for the PCR and specific ZIP-codes (ZIPChute probe). As one of the primers was biotinylated, the biotinylated amplicon was captured onto a streptavidin coated surface after the PCR. These ZIPs contained a unique sequence that enabled their size differentiation during electrophoresis. This assay allowed the simultaneous detection of potentially up to 48 DNA sequences (endogenous, element-, construct-, and event-specific targets). In their paper simultaneous detection for up to seven targets was shown with a detection limit range of 0.1-1%.

Peano et al. [16] also applied separate probes and the ligation detection reaction was combined with a universal array approach. They performed an extra pre-amplification step before the ligation and describe the detection of five different GMOs when present at 0.4% each, relative to non-GM (conventional) material.

Ligation based systems have been used for other nucleic acid experiments as well. Ericsson et al. [17] used a dual-tag microarray platform for high-performance nucleic acid analysis. After the dual-tag probe ligation, solution phase rolling circle amplification was performed and the detection was carried out on chip. Different other multiplex approaches have been described by several authors [18-23] to detect GMOs but none of the techniques have so far shown more multiplicity than the ligation detection methods.

**Figure 2:**
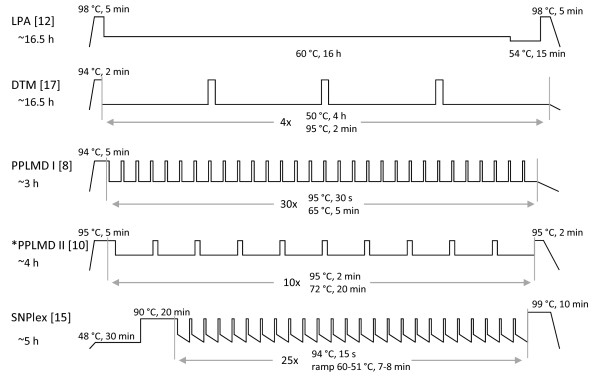
**Differences among the published ligation protocols**. For each method, a temperature (y-axis) × time diagram (x-axis) is shown as it was used in the indicated references. LPA: Ligation-dependent Probe Amplification, DTM: Dual Tag Microarray, PPLMD: Padlock Probe Ligation in combination with Microarray Detection. *PPLMD II: Edwards et al. [10] did not use any abbreviated protocol name for their method contrary to the other authors [8,12,15,17], but for clarity this protocol was called PPLMD II in this article.

It was noted that the above-mentioned ligation based methods used various ligation protocols, which are radically different from each other with regard to temperatures, incubation times and number of reaction cycles (Figure 2). There are also some differences among the types of probes, probe concentrations and the type of ligation enzymes, but all publications seem to reach similar sensitivities so far. In the literature different kinds of PCR parameters have been described as well, but the different PCR parameters are not likely to have so much effect on the sensitivity compared to the ligation step. The large differences among these ligation procedures led us to compare different protocols in a common sample setting to try and find the factors that are most important for specific and sensitive GMO (multi)detection.

To this end the present study aimed to compare different ligation protocols including reaction temperatures with the PLP system in different GM mixes. Three of the selected ligation protocols were GMO detection related [8,12,15] and the other two were used for other types of nucleic acid analysis [10,17]. The detection was performed with TaqMan probes designed for PLPs in all cases, using the same real-time PCR parameters in fourplex and the best results were confirmed on microarray. Further aim was to test the specificity of the system using the best ligation protocol, based on the results of the ligation comparison. Finally, the transferability of method was tested in an interlaboratory exchange study as an initial validation step of the approach.

## Methods

### Plant materials

For detailed information on the composition of the genetically modified organisms (GMOs) used for the experiments, see the GMO Detection Method Database [24] and GM Crop Database [25]. Ground seed materials were purchased from IRMM (Geel, Belgium) and AOCS (Urbana, IL, USA) (Table 1). The same reference materials were used throughout the study and the results reflect these materials. As such, differences in any of the GMOs that may occur in different years of cultivation are not part of the present study.

**Table 1:** Final composition of the prepared GM mixes

Experiment	Name	Component	Source	Code	Final concentration*	GMO construct copy numbers in 200 ng DNA**
Ligation comparison	GM mix 1	1% Bt176 maize	IRMM	ERM-BF411d	0.1% Bt176	18
		10% TC1507 maize	IRMM	ERM-BF418d	5% TC1507	889
		0% MON810 maize***	IRMM	ERM-BF413a		
	GM mix 2	5% Bt176 maize	IRMM	ERM-BF411f	2.5% Bt176	444
		10% TC1507 maize	IRMM	ERM-BF418d	1% TC1507	178
		0% MON810 maize***	IRMM	ERM-BF413a		
	GM mix 3	100% Bt176 maize	RIKILT		2.5% Bt176	444
		100% TC1507 maize	RIKILT		2.5% TC1507	444
		0% GTS 40-3-2 ****	IRMM	ERM-BF410a		

Specificity testing	Mix 1	> 97.9% 281-24-236x3006-210-23 cotton	IRMM	ERM-BF422b	16.7%	6789
		100% LL25 cotton	AOCS	0306-D	16.7%	6789
		100% RF3 canola	AOCS	0306-G	16.7%	13047
		100% LL62 rice	AOCS	0306-I	16.7%	37111
		> 89.9% MON863xNK603xMON810 maize	AOCS	0406-C	16.7%	2966
		> 99.4% MON15985xMON1445 cotton	AOCS	0804-F	16.7%	6789
	Mix 2	oat	Biolytix		33.3%	
		barley	Biolytix		33.3%	
		wheat	Biolytix		33.3%	

Exchange study	1% GM mix	5% Bt176 maize	IRMM	ERM-BF411f	1% Bt176	178
		10% TC1507 maize	IRMM	ERM-BF418d	1% TC1507	178
		10% MON863 maize	IRMM	ERM-BF416d	1% MON863	178
		0% MON810 maize***	IRMM	ERM-BF413a		
	0.1% GM mix	1% Bt176 maize	1% GM mix		0.1% Bt176	18
		1% TC1507 maize	1% GM mix		0.1% TC1507	18
		1% MON863 maize	1% GM mix		0.1% MON863	18
		0% MON810 maize***	IRMM	ERM-BF413a		
	0% GM mix	0% MON810 maize***	IRMM	ERM-BF413a		

*expressed as percentage of total mass

**based on information in the reference material certificate, unless stated otherwise in the certificate, GMO constructs were assumed to be present as single copy, homozygous insertions in the native genomic DNA

***certified as < 0.2% MON810

****certified as < 0.3% GTS 40-3-2, commercially known as Roundup Ready soy.

### DNA extraction

The following protocol was used for all maize samples except for 100% Bt176 and 100% TC1507. Plant material (100 mg), 150 μl MilliQ treated water (MQ) and 350 μl CTAB extraction buffer (20 g/l CTAB; 1.4 M NaCl; 0.1 M Tris-HCl; 20 mM EDTA) was mixed together with 5 μl RNaseA (Qiagen) and incubated for 15 min at 65°C. Subsequently, 20 μl 20 mg/ml Proteinase K (Fermentas Molecular Biology, Germany) was added and the mix was incubated for 15 min at 65°C. After adding 200 μl of Buffer AP2 (Qiagen DNeasy Plant Minikit) the mix was placed on ice for 5 min. Further steps continued from step 4 of the Qiagen DNeasy Plant Minikit protocol without modifications [26]. For GTS 40-3-2, 100% Bt176 and 100% TC1507 the DNA Wizard Clean up system for genomic DNA (Promega) was used. Plant material (200 mg) was weighed and DNA extraction was performed according to Zimmermann et al. [27]. DNA concentrations and the purity of the DNAs (A260/A280 and A260/A230) were measured with NanoDrop spectrophotometer (NanoDrop ND-1000, V3.5.2).

### Padlock probes/PLPs

Different mixtures of PLPs were prepared for the different purposes. The mixture used for the ligation comparison contained the PLPs for detection of *cry1Ab, bar*, TC1507 and maize endogenous *hmg *in concentrations shown in the protocol section.

A 20-plex PLP mixture was used for testing the specificity and consisted of the following PLPs: three event specific PLPs (GTS 40-3-2, MON810, Bt176), seven element specific PLPs (*cry1F, pat, bar, CP4-epsps*, p35SCaMV, p35SFMV and *barstar*), one construct specific (Bt11), eight species specific (maize, soy, cotton, rice, canola, wheat, oat and barley) and one control PLP (spikelock). Every PLP was present at a concentration of 250 pM.

For the exchange study a tenplex PLP mixture was prepared containing maize *hmg*, maize *zein*, p35SCaMV, *pat, bar, cry1F, cry1Ab, cry3Bb*, TC1507 and the Spikelock, also at a final concentration of 250 pM per PLP.

The relevant sequences of the newly designed PLPs are given in Table 2 and Table 3. The same primer binding sites were used as previously published [8]. All other PLPs were published before [8,9].

**Table 2:** DNA sequences of the oligonucleotides used in padlocks

Name	Type	T1, 5' target (5'-3')	cZIP sequence (5'-3')	T2, 3' target (5'-3')	Size (nt)
TC1507	event	CGCGGTTTGTGATATCGTTAACCATTACATTGAGACGTCTAC	ATGATGTGCAAAGTGCCGTC	CTTTCGTTCTTGTGTTC	126
*pat*	element	CAACCACAGACTTAAAACCTTGCGCCTCCATAGAC	ACGCTAATGACGGCAGTGCA	GGAAGGCCTATAACAG	118
*cry1F*	element	GAAACGTGTAAGGGACAGGGAGATGTCTAACGGCAATC	ATTTGACGAACGTATGCCGC	ACAAACTCAGACAACAG	122
*cry1Ab*	element	CAGGTTGGTGCACTTGGTGAGGGGGATCTGGGTGATTTGG	ACATCCTGGACACGAGTGAC	GGTGCCGCTGCC	119
*cp4-epsps*	element	GGCCTTGCCCGTATTGATGACGTCCTCGCC	ATTAACTCGACTGCCGCGTG	CCCATGGCCTGCAT	111
*barstar*	element	GCCTCCATTCCAAAACGAGCGGGTACTCCA	TCCTCTCGTTGGATGTGAGC	CTTGCTTTGTTCAAACT	114
Bt11	construct	ATCTTCGCTAGAGTAAGGGTTTCTTATATGCTCAACACATGAGCG	GAATGCGGTTCAACAGTCTT	GCGAGGTGAAGAGG	126
maize (*hmg*)	species	CACACAAACGCACGCGTAAAACAATTAATCAGCACGAG	CTGCGGTGTCAGTGATCTCT	GCCTTGTCCTACAATC	121
maize (*zein)*		CTGTGGCATCATCACTGGCATCGT	GTACTACATTCGTGCGATGG	TTAGGCGTCATCAT	124
rice	species	CCATTGCTGTCTCTGCAAGCTCACGCGC	ATGCAGCGTAGGTATCGACT	CGGCAGCAACTCTCA	110

**Table 3:** Sequences of TaqMan probes designed for PLPs

Name	Reporter dye-5' sequence 3'-quencher	Amplicon size (nt)
maize (*hmg*)	Cy5-TGCGGTGTCAGTGATCTCTGCCTTGTCCT-BHQ2	121
*bar*	TR-TGCTCCGTGCGAAATATGACCGTGCTT-BHQ2	112
TC1507 event	FAM-AAGTGCCGTCCTTTCGTTCTTGTGTTCCG-BHQ1	126
*cry1Ab*	VIC-ACACGAGTGACGGTGCCGCTGCC-BHQ1	119

TR: Texas Red, BHQ: Black Hole Quencher.

### Ligation protocols

All ligation reactions were performed in a BioRad iCycler IQ 3.021.

#### *According to Prins et al*. [8] (PPLMD I)

Two hundred nanograms of DNA was used for the ligation reaction which consisted of 1 × *Pfu *ligation buffer (Stratagene); 12% PEG6000 (Fluka, Germany); 0.1 U/μl *Pfu *ligase (Stratagene) and 25 pM of each PLP in a final volume of 10 μl. The following cycle conditions were used 94°C for 5 min; 95°C 30 s, 65°C 5 min for 30 cycles.

#### *According to Ericsson et al*. [17] (DTM)

Ligation reactions were performed in 10 μl comprising 0.1 nM of each PLP, 200 ng DNA, and 5 U of Ampligase in Ampligase buffer (Epicentre Biotechnologies, WI, USA). The ligation reaction was performed for 4 cycles of 4 h at 50°C and 2 min at 95°C.

#### *According to Ehlert et al*. [12] (LPA)

DNA (250 ng) was denatured for 5 min at 98°C. Subsequent 1.5 μl of MLPA buffer (MRC Holland, Amsterdam, the Netherlands) and 1.5 μl of a mixture of 2 fmol of each PLP were added and the reaction was kept for 16 h at 60°C. The subsequent ligation reaction was performed at 54°C for 15 min by adding 3 μl Ligase-65 buffer A, 3 μl Ligase-65 buffer B, 25 μl MilliQ and 1 μl Ligase-65 (MRC-Holland, Amsterdam, the Netherlands). A final incubation for 5 min at 98°C was used to inactivate the enzyme.

#### *According to Edwards et al*. [10] (PPLMD II)

DNA (25 ng) was mixed with 1 μl of a 300 pM PLP mix, 1 U of Ampligase (Epicentre, Madison, Wisconsin, USA) and 3 μl of Ampligase reaction buffer in a total volume of 30 μl. The following cycling conditions were used: 95°C for 5 min; 95°C for 2 min and 72°C for 20 min for 10 cycles and enzyme inactivation at 95°C for 2 min.

#### *According to Chaouachi et al*. [15] (SNPlex)

Ligation chemicals were applied as described by Prins et al. [8]. Cycling conditions were used according to Chaouachi et al. [15] and consisted of the following steps: 48°C 30 min; 90°C 20 min; after 94°C 15 s, 60°C 30 s, 51°C with a 3% ramp of 30 s for 25 cycles; and 99°C 10 min.

### PCR detection

In all cases Linear After The Exponential (LATE)-PCR [28] in combination with asymmetric primer concentrations [8,9] was used in order to create single stranded PCR products. For the ligation comparison detection was performed using real-time PCR. TaqMan probes were designed with the aid of Beacon designer 7.0 Software (Premier Biosoft, California, USA). All primers and probes were purchased from Biolegio, the Netherlands. Sequences of the TaqMan probes are described in Table 3.

All real-time PCRs were performed on a BioRad iCycler IQ with Universal Mastermix No-ROX PCR kit from Diagenode (Liège, Belgium). Reaction tubes contained 4 μl ligation mixture, 12.5 μl mastermix, 500 nM forward primer, 50 nM reverse primer and 400 nM TaqMan probe in a total volume of 25 μl. The following cycling protocol was used: 10 min at 95°C followed by 40 cycles consisting of 10 s at 95°C and 40 s at 60°C.

### Microarray analysis

For PLP specificity testing as well as the exchange study, detection was performed on a microarray using a Cy3 labelled reverse primer in the LATE-PCR as described by Prins et al. [8]. Two brands of arrays were used for the experiments. The EAT (Eppendorf Array Technologies, Belgium) slide contained 8 microarrays and the Isogen (Isogen, the Netherlands) slide contained 2 microarrays. In both cases each array contained 100 spotted ZIP-codes (20-mer oligonucleotide sequences from Affymetrix) with a 10-mer A-tail (and C6 to linker) in quadruplicate per microarray. 2 μl denatured labelled mix was applied to either 38 μl (EAT array) or 63 μl (Isogen array) hybridisation mixture. 0.4 nM (EAT array) or 0.2 nM (Isogen array) Cy3 labelled cZIP-B3 was used as a hybridisation control. Following steps were performed according to Prins et al. [8].

### Data analysis

#### Real-time PCR

Four observations for the optimal temperature for each method were subjected to ANOVA (α = 0.01) and Tukeys HSD post-hoc testing for statistical evaluation of the results. Results were expressed as the ΔCt, i.e. the difference between the Ct value of the sample and the Ct value of 0% GTS 40-3-2 in the same experiment. Only samples with at least three replicates of positive ΔCts were taken into account.

#### Microarray experiments

For the comparison of the different ligation methods the outliers and obvious artefacts were removed manually. Density values (Dens) of the spots were used for the further analysis. A two tailed t-test was used to evaluate differences between the two methods for two separate experiments.

In case of the examination of specificity of the 20 probes the outliers and obvious artefacts were also removed manually. The samples were scored positive or negative on the basis of visual inspection of the array scans.

In case of the transfer project the outliers were filtered on the basis of the relative SD of the spot signals, the percentage at ceiling and the interquartile range (IQR). Outliers were defined as values above: Q3+3(IQR) or below: Q1-3(IQR). Further data analyses were done according to Prins et al. [8]. Raw data for the microarray experiments are available as additional files 1 and 2 as Comparison protocols.csv and Transfer.csv.

## Results

### Ligation comparison: real-time PCR analysis

Five different published ligation detection protocols were tested on three DNA mixes with different GM targets, 0% GTS 40-3-2 DNA was used as a negative control in all experiments. The methods were the padlock probe ligation in combination with microarray detection (PPLMD I) [8], another padlock probe approach also combined with microarray detection (PPLMD II) [10], the protocol used in the dual tag microarray method (DTM) [17], a ligation-dependent probe amplification (LPA) protocol [12] and the SNPlex approach as described by Chaouachi et al. [15]. The PLPs that were used were specific for the maize endogenous *hmg *gene, the maize GM elements *cry1Ab *and *bar *and the maize GM event TC1507. TC1507 maize was used as a source for the TC1507 event target and Bt176 maize as a source for the *bar *and *cry1Ab *element targets. Subsequent PCR amplification and identification were the same for all comparisons just as the four padlock ligation probes used in all protocols, in order to only investigate the influence of the specific detection part of the different protocols. Instead of microarray identification of amplified products after the PCR, the system was adapted for real-time detection. For this purpose, TaqMan probes labelled with different fluorescent dyes were designed specifically for the four different PLPs. The TaqMan probes were designed on the so-called short arm, between the unique target site 2 and cZIP regions for the four PLPs. Except for the *bar *PLP, these PLPs were not published before. Before using the newly developed PLPs in the ligation comparison study, they were tested for general performance based on previously published criteria [8]; their circularizing capacity was tested on single stranded synthetic targets as well as genomic DNA using SYBR green PCR. In all cases, the PLPs showed Ct values at least 4 cycles earlier for their specific target than for the non-target control. After this each PLP was examined in simplex with microarray analysis to screen for possible cross-hybridizations with other ZIP-codes on the array; none were observed (data not shown).

In a first round of ligation comparisons, both the ligation chemicals and reaction times were according to the published methods. In this case, the SNPlex protocol could not be evaluated as the precise buffer compositions were not disclosed by the authors or the company [29]. On top of that, at least at the time of the experiments, the buffer was not sold separately, but only as part of the rather expensive full SNPlex kit. For each of the remaining four methods, a range of ligation temperatures was tested. The range was different for all methods and included eight distinct temperatures including the published temperature and the Tm of the PLPs. A summary of the implemented ligation comparisons is shown in Table 4. Subsequent PCR was performed in duplicate. After evaluation of the results, the comparison was repeated with three distinct temperatures, one above and one below the optimal temperature of the previous experiment. The optimal temperature was confirmed in all cases. For the LPA protocol the ligation procedure consisted of two distinct steps, hybridization and ligation. Based on their paper, the hybridization step was the most critical, therefore the temperature range was tested for the hybridization step while all ligations were performed at the published temperature of 54°C. Results are shown in Table 5. The PPLMD I protocol performed statistically best for the *hmg *detection, while both the PPLMD I and DTM protocols performed best for the *cry1Ab *and *bar *detection; TC1507 detection was unsuccessful with all four protocols.

**Table 4:** Differences among ligation methods used for the ligation comparison

		Ligation temperature (°C)	
		
Chemicals	Cycle program	Tested range	Published
DTM [17]	DTM	46-65*	50
PPLMD II [10]	PPLMD II	59.3-75*	72
LPA [12]	LPA	H:51.7-65*; L:54	H:60 / L:54
PPLMD I [8]	PPLMD I	53-68*	65

PPLMD I	DTM	50; 55.5; 59.3; 65	50
	PPLMD II	61; 65; 67.8; 72	72
	LPA	H:60 / L:54	H:60 / L:54
		H:71 / L65	
		H:65 / L:59	
		H65 / L:65	
	SNPlex [15]	Ramp 1: 60-51	3% Ramp: 60-51
		Ramp 2: 74-65	
		Ramp 3: 70-61	

* Range with eight different temperatures. H: hybridisation, L: ligation

**Table 5:** Results of the ligation comparison using different chemicals with different cycle conditions

			ΔCt (SD)				
				
Target	GM mixes	Target %	PPLMD I - 65°C	DTM - 63.6°C	LPA - 62.4°C	PPLMD II - 62.5°C	p-value ANOVA
*hmg*	1	100	14.3 (0.4)^a^	8.1 (0.7)^b^	6.9 (0.4) ^bc^	3.8 (3.8) ^c^	5.7E-05
	2	100	13.8 (0.9) ^a^	8.1 (0.3) ^b^	6.4 (0.4) ^bc^	3.3 (3.5) ^c^	2.6E-05
	3	5	7.8 (1.6)	ND	ND	ND	-

*bar*	1	0.1	ND				-
	2	2.5	3.9 (0.5)	4.5 (2.3)	1.0 (0.4)	ND	> 0.01
	3	2.5	3.5 (1.2) ^ab^	4.8 (2.3) ^a^	0.8 (0.6) ^b^	0.6 (0.5) ^b^	2.3E-03

*cry1Ab*	1	0.1	ND				-
	2	2.5	7.5 (0.0) ^a^	8.2 (0.1) ^a^	3.2 (1.0) ^b^	0.5 (0.2) ^c^	1.1E-10
	3	2.5	6.9 (0.5) ^b^	8.3 (0.7) ^a^	3.4 (0.6) ^d^	0.6 (0.4) ^d^	6.3E-10

TC 1507	1	5	ND				-
	2	1	ND				
	3	2.5	ND				

SD: standard deviation of the ΔCt values

Values marked with different superscript characters indicate groups (for the same target-mix combination) that are significantly different according to Tukeys HSD test (P < 0.05); x^a ^is different from y^b^, z ^ab^is not different from either.

ND: not detected or negative ΔCt.

The temperature shows the optimal temperature from which the data were analysed.

In a second round of comparisons only the reaction times were kept as in the published protocols. Buffers and chemicals were all according to the PPLMD I protocol. For this series four ligation temperatures were evaluated for the PPLMD II and DTM protocols. Three hybridization temperatures and three ligation temperatures were combined to yield four different combinations for the LPA protocol. For the SNPlex protocol three temperature ramp ranges were tested. PCRs were again performed in duplicate and the whole experiment was repeated to yield four observations per sample. Analysis was performed as for the first round of comparisons and the results are shown in Table 6. The LPA, DTM and PPLMD II protocols performed better in this comparison than in the first one. In this comparison the PPLMD I protocol performed best for *hmg *in GM mix 1 and 2, while for mix 1 the SNPlex and for mix 2 the PPLMD II protocol performed statistically the same as the PPLMD I protocol. For the *cry1Ab *test the PPLMD II protocol showed the best results. For *bar *detection, no significant differences were found between methods, and TC1507 detection was again unsuccessful.

**Table 6:** Results of the ligation comparison using chemicals from PPLMD I protocol with different cycle conditions

			ΔCt (SD)					
				
Target	GM mixes	Target %	PPLMD I 65°C	DTM 65°C	LPA H60-L 54°C	PPLMD II 65°C	SNPlex Ramp 70-61°C	p-value ANOVA
*hmg*	1	100	14.3 (0.4) ^a^	10.0 (0.7) ^c^	9.0 (1.4) ^c^	12.0 (0.2)^b^	12.6 (0.7)^ab^	1.2E-06
	2	100	13.8 (0.9) ^a^	10.5 (1.0) ^cd^	9.3 (0.9) ^d^	12.6 (0.7) ^ab^	12.0 (0.2) ^bc^	6.5E-06
	3	5	7.8 (1.6)	5.2 (1.3)	4.7 (2.2)	7.5 (1.1)	7.1 (0.2)	> 0.01

*bar*	1	0.1	ND					
	2	2.5	3.9 (0.5)	2.5 (0.7)	4.5 (3.19)	4.0 (0.7)	3.2 (1.0)	> 0.01
	3	2.5	3.5 (1.2)	2.5 (1.3)	3.4 (3.5)	3.3 (1.3)	3.9 (0.7)	> 0.01

*cry1Ab*	1	0.1	ND					
	2	2.5	7.5 (0.0) ^b^	5.2 (0.5) ^b^	4.4 (2.5) ^b^	11.4 (0.6) ^a^	7.5 (2.3) ^b^	1.2E-04
	3	2.5	6.9 (0.6) ^b^	5.7 (0.5) ^b^	5.4 (2.4) ^b^	10.9 (0.3) ^a^	7.5 (2.1) ^b^	5.7E04

TC 1507	1	5	ND					
	2	1	ND					
	3	2.5	ND					

SD: standard deviation.

Values marked with different superscript characters indicate groups (for the same target-mix combination) that are significantly different according to Tukeys HSD test (P < 0.05); x^a ^is different from y^b^, z^ab ^is not different from either.

ND: not detected or negative ΔCt.

The temperature shows the optimal temperature from which the data were analysed.

### Ligation comparison: microarray analysis

**Figure 3:**
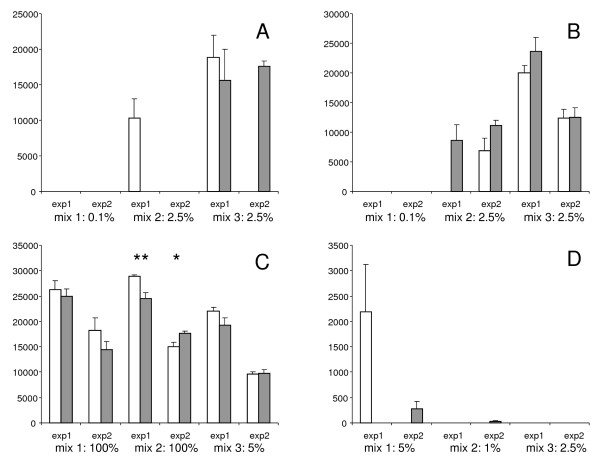
**Results of the comparison of the two best methods on microarray**. The mean value and standard error of four individual spots on a microarray are expressed in arbitrary fluorescence units on the y-axis. On the x-axis the number of the experiment, the number of the DNA mix and the weight percentage of the genomic DNA is given for A: *bar*, B: *cry1Ab*, C: *hmg *and D: TC1507 detection. * indicates a p-value of < 0.05 and ** a p-value of < 0.01 in a two-tailed student's t-test between the PPLMD II protocol in white bars and the PPLMD I protocol in grey bars.

The PPLMD I and PPLMD II protocols were also compared using microarray detection, as these two performed overall best in the real-time comparison. For microarray analysis, four observations were analysed per sample, as on each array each cZIP probe was spotted in quadruplicate. Eight such arrays were spotted on a glass slide and in one experiment the three GM mixes and the 0% GTS 40-3-2 control sample were tested for both protocols on one slide. The whole experiment was repeated with a second slide. Positive signals were defined as signals with an observed mean fluorescence significantly higher than that of the control sample. Especially for *bar *detection high background values were observed in the control sample (data not shown). For TC1507, positive signals were observed in some cases, contrary to the real-time detection. Like for real-time analysis, no consistent significant difference between the two protocols was observed. In fact, only for the *hmg *detection significant differences were observed in GM mix 2. In the first experiment the PPLMD II protocol performed better while in the second experiment the PPLMD I protocol performed better. Results of the comparison of the two best methods are shown in Figure 3.

### Specificity of the PLPs

Further aim was to test the specificity of the PLPs using one of the best ligation protocols (PPLMD I) in combination with microarray detection. A selection of 20 PLPs was chosen for this, including six more PLPs that were not published before (Table 2). After checking the general performance of the PLPs as described under the ligation comparison, they were tested further in multiplex reactions. For this purpose, two DNA mixes were prepared with different targets. The GM mix contained equal amounts of DNA of different GM reference material, containing at least 90% GM material each. This mix contained targets for four endogenous genes, seven GM elements, one event and one for the internal control. The cereal mix contained equal amounts of 100% oat, barley and wheat DNA. Detailed information is shown in Table 7. The PLPs were mixed together and were tested on both mixes. In the GM mix all of the 13 targets (including the internal control) were detected and the seven probes, for which no targets were present, were negative as expected (Table 7). In case of the cereal mix three out of four positive targets were detected and the others were negative. So, except for the barley probe, all of the tested PLPs showed a positive signal on the array.

**Table 7:** Results of 20 probes tested in two mixes

		GM mix		Cereal mix	
Target	Type	Expected results	Actual results	Expected Results	Actual results
GTS 40-3-2	GM event	**-**	**-**	**-**	**-**
MON810 event	GM event	**+**	**+**	**-**	**-**
Bt 176	GM event	**-**	**-**	**-**	**-**
p35SCaMV	GM element	**+**	**+**	**-**	**-**
*cry1F*	GM element	**+**	**+**	**-**	**-**
*pat*	GM element	**+**	**+**	**-**	**-**
*bar*	GM element	**+**	**+**	**-**	**-**
*cp4-epsps*	GM element	**+**	**+**	**-**	**-**
p35SFMV	GM element	**+**	**+**	**-**	**-**
*barstar*	GM element	**+**	**+**	**-**	**-**
Bt11	GM construct	**-**	**-**	**-**	**-**
maize *(zein)*	species	**+**	**+**	**-**	**-**
soy	species	**-**	**-**	**-**	**-**
cotton	species	**+**	**+**	**-**	**-**
rice	species	**+**	**+**	**-**	**-**
canola	species	**+**	**+**	**-**	**-**
wheat	species	**-**	**-**	**+**	**+**
oat	species	**-**	**-**	**+**	**+**
barley	species	**-**	**-**	**+**	**-**
spikelock	control	**+**	**+**	**+**	**+**

### Detection level and transferability

A tenplex PLP system in maize was tested regarding the detection level and the transferability of the method to a different laboratory. The transfer experiments were carried out following the PPLMD I protocol. The NIB served as transfer laboratory.

Three GM mixes were tested containing 0% (negative control), 0.1% and 1% GM material. The 0.1% and 1% GM mixes contained targets for six GM elements, two plant-species, one GM event and one internal control. The same ten PLPs were mixed together and tested on the three GM mixes, see the details in Table 8. MON810, 0% was used as a negative control. Samples were coded randomly prior to sending. The results were re-encoded by the transfer lab prior to sending in the raw data. After data analysis and exchange the codes were broken. In the test laboratory results showed positive signals in each case for the 1% and 0.1% GM material apart from the TC1507 event. In case of the 0% GM mix only the PLPs for endogenous targets showed positive signals, no false positive signals were observed (Table 8).

**Table 8:** Results of the tenplex system tested on different GM mixes by RIKILT and by NIB

		1% GM mix		0.1% GM mix		0% GM mix	
Target	Type	RIKILT	NIB	RIKILT	NIB	RIKILT	NIB
maize (*hmg*)	species	**+**	**+**	**+**	**+**	**+**	**+**
maize (*zein*)	species	**+**	**+**	**+**	**+**	**+**	**+**
p35SCaMV	GM element	**+**	**+**	**+**	**+**	**-**	**-**
*pat*	GM element	**+**	**+**	**+**	**+**	**-**	**-**
*bar*	GM element	**+**	**+**	**+**	**-**	**-**	**-**
*cry1F*	GM element	**+**	**+**	**+**	**+**	**-**	**-**
*cry1Ab*	GM element	**+**	**+**	**+**	**+**	**-**	**-**
*cry3Bb*	GM element	**+**	**+**	**+**	**+**	**-**	**+/-**
TC1507	GM event	**-**	**-**	**-**	**-**	**-**	**-**
spikelock	control	**+**	**+**	**+**	**+**	**+**	**+**

**Figure 4:**
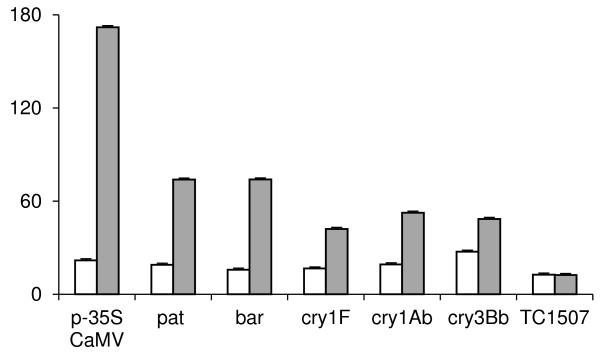
**GM related microarray results of test laboratory for the 1% GM mixture**. The y-axis represents the mean pixel density, normalized for *hmg*. On the x-axis the GM related targets are given. Out of the seven GM related targets, only TC1507 event did not show significant signal compared to the control slide. The results of the control slide are shown in white bars and grey bars represent the target slide.

GM related signals of the test laboratory (RIKILT) for the 1% GM mixture are shown in Figure 4 as an example. Out of the seven GM related targets, only TC1507 event did not show significant signal compared to the control (MON810, 0%). In case of the two endogenous genes (*hmg, zein*) similar results were observed on the target and control slide as expected and the adequacy of the ligation was proven with the internal control (data not shown). Signals were normalised for the *hmg *target signal.

The experiments were carried out by the transfer laboratory (NIB) twice on separate days. During the transfer of the method from four samples results were obtained that could be analysed: two 0% samples, one 0.1% sample and one 1% sample, the other two samples suffered from technical errors (Table 8). In case of the 0% samples, only the *cry3Bb *spot was scored as a false positive once, the other spots showed the expected results, namely the GM related spots were all negative and the endogenous spots were positive. Of the 1% sample, one false negative signal was observed for TC1507, all other spots were positive. For the 0.1% sample both endogenous spots were positive as were five of the seven GM spots, no significant signals were observed for *bar *or TC1507.

## Discussion

So far, various ligation protocols, very different from each other, have been published for the purpose of specific multiplex DNA detection. In this study, performance of a number of protocols was compared using identical probes and samples. For all comparisons, a real-time PCR strategy was used for signal detection. After choosing the best protocol, the specificity, detection level and the transferability of the method were tested using microarrays.

The ligation protocols were compared in two rounds of experiments. In the first round, both chemicals and reaction times were kept the same as the published methods. In a second round, only the reaction times were kept as in the published protocols while the chemicals were all according to the PPLMD I protocol. In both cases ligation temperature ranges were tested as well. The best temperatures were chosen in each case (Table 5, 6), which was always between 60 and 65°C. At lower temperatures signals in non-target reactions increased, in some cases to the same level as observed for specific reactions. At higher temperatures increased Ct values were observed, indicating less efficient ligation reactions. At the chosen best temperature for each protocol significant differences were found among the different ligation techniques. The PPLMD I and PPLMD II protocols performed overall best in the real-time comparison after two rounds of ligation comparisons. In both rounds the LPA protocol resulted in atypical amplification curves in most cases. A possible cause for this could be that this method was designed to work with two separate "bipartite" ligation probes contrary to the PLP system which was used in this paper. The PPLMD I, PPLMD II and SNPlex methods were most similar to each other providing similar results whereas with the LPA and DTM protocols, later Ct values were observed in the PCR, indicating a less efficient prior ligation reaction. The differences might be explained by the fact that these two protocols have just a few long cycles contrary to the others which consist of more short cycles (Figure 2). Furthermore, these two protocols were not designed or optimized for the low level detection demands in GMO detection but were originally used for other types of DNA targets. Our results confirmed the importance of choosing the best ligation protocol for a certain ligation based system in order to reach the appropriate specificity and detection level.

The two best protocols (PPLMD I and PPLMD II) were also compared using microarray detection to confirm the results of the ligation comparison. Like for the real-time PCR analysis no consistent significant difference was observed between the two protocols. The only difference between the array and real-time detection was the positive signals observed for TC1507 event in some cases on array contrary to the results of the real-time detection.

For further experiments the PPLMD I protocol was chosen to test the specificity, sensitivity and the transferability of the method. A combination of 20 PLPs was selected to test the specificity in a complex matrix of plant DNAs isolated from GM or non GM reference materials. Our results showed that all but one probe reacted specifically with their targets when present, and the ones for which no target was present showed negative results. In total, 13 targets were detected in a single multiplex reaction. According to the literature the SNPlex assay [15] allows the simultaneous detection of up to 48 DNA sequences (endogenous, element-, construct- and event-specific targets), but in their article up to sevenplex detection was actually shown. Different other multiplex approaches have been described by several authors, but none of the techniques have shown higher multiplicity in GMO detection than the results presented here.

Another very important factor in GMO detection is the detection level and the transferability of the method. To examine these parameters a tenplex PLP system was tested in a test laboratory as well as in a transfer laboratory. During the comparison of protocols, the 0.1% signals for bar and Cry1Ab were scored negative due to the background in the 0% GTS 40-3-2 reference material that was used as negative control. It has been reported in literature, as well as on certificates of certified reference materials that 0% CRMs may contain a certain low level of the GMO they are supposed to be negative for as well as other GMOs [30,31]. Furthermore, it is not very probable, given the shown specificity of PLPs in the present and previous papers [8,9], that the signal in the 0% GTS 40-3-2 was due to cross-reaction. For these reasons, a different reference material was selected as negative control in the transfer experiments, particularly 0% MON810. During the experiments a detection level down to 0.1% was reached for most of the GM targets while the endogenous genes (*hmg, zein*) were present at 100% level, as the mix contained only maize material. Moreover, similar results were achieved by the two laboratories indicating a good transferability and robustness of the method. The detection level that was reached is sufficiently lower than the 0.9% labelling obligation which has been defined according to the EU regulation [32] and is indeed comparable to the detection level that is now considered adequate for single GMO detection methods. Especially in the transfer study it was shown that a 0.1% level could be reached for most genomic targets which would be in line with the novel EU regulation 619/2011, which sets a technical zero of 0.1% for low level presence of GMOs pending authorisation in the EU while having been approved elsewhere [33]. The detection level stated in this paper reflects the lowest reproducibly detected level in this study, as such it is not a fully validated limit of detection (LOD) as required for methods for legal purposes. Such a full validation is part of future experiments. The weight percentage of a GMO reference material can be translated to an estimation of GMO related copy numbers. For instance, in TC1507, the 200 ng input of a 0.1% sample would contain approximately 18 copies, assuming a heterozygous single insertion. Because of the differing genetic composition of different parts of the seeds of monocotyledons (e.g. maize endosperm, seed coat and embryo), the value of the DNA ratio in the reference material may be not the same as the value of the certified powder mass fraction [34].

During the ligation comparison and also during the transfer of the method problems were observed with TC1507 event and *bar *detection. These two probes performed well in initial simplex evaluation but showed suboptimal results in a multiplex situation. Still they were included throughout the study on purpose. This indicates the necessity of fine-tuning the parameters for optimal probe design. This aspect requires further attention in future experiments.

## Conclusions

The outcome of this study demonstrated that some ligation protocols are more effective than others, but at the same time that different protocols can lead to similar results. Secondly, the applied PLP system using the optimal ligation protocol was able to identify more GMO related DNA targets simultaneously than previously published and had a detection level down to 0.1% for six GMO element targets. The reproducibility of this approach was also shown in a transfer laboratory. Further experiments and validation are necessary for the method in order to implement this elegant procedure in the routine analysis of food and feed samples.

## Authors' contributions

GU performed the comparison of the protocols, coordinated the transfer study, performed the transfer study at RIKILT and drafted the manuscript, JPvD supervised all practical and statistical work, drafted and finalized the manuscript, TWP performed sequence analysis, probe design and helped drafting the manuscript, MMV performed the specificity testing and drafted the manuscript, AMAVH assisted in experimental work and helped drafting the manuscript, HGB assisted in experimental work and helped drafting the manuscript, DM performed the transfer study at NIB and helped drafting the manuscript, KG coordinated the transfer study at NIB, EJK coordinated the study and drafted the manuscript. All authors read and approved the final manuscript.

## Supplementary Material

Additional file 1 **Comparison protocols.csv**. raw data for the microarray spots and the results of the statistical analysis of the comparison of the different ligation protocols. Click here for file

Additional file 2 **Transfer.csv**. raw data for the microarray spots and the results of the statistical analysis the transfer of the PPLMD method. Click here for file
